# Control of Joint Reaction Forces During Single-Joint Strengthening Exercises via Adaptive Electromechanical Technologies: An Analytical Biomechanical Framework

**DOI:** 10.3390/bioengineering13030270

**Published:** 2026-02-26

**Authors:** Andrea Biscarini

**Affiliations:** Department of Medicine and Surgery, University of Perugia, 06132 Perugia, Italy; andrea.biscarini@unipg.it; Tel.: +39-075-585-8135

**Keywords:** biomechanics, rehabilitation, adaptive control, joint loading, knee, ACL

## Abstract

**Background**: Modern electromechanical technologies can be integrated into strength training machines to regulate the magnitude, direction, and point of application of resistance during exercise, either through preprogrammed settings or adaptively in response to real-time kinematic data. However, this potential remains largely unexplored. The objective of this study was to investigate how these new-generation devices may be managed to enable precise control of the mechanical load applied to specific joint structures during strengthening exercises. **Methods**: A foundational framework of biomechanical equations was developed to establish the functional relationships between joint reaction forces and key variables, including kinematic parameters (joint angle, angular velocity, and angular acceleration) and resistance characteristics (magnitude, direction, and point of application). The analysis focused on analytically determined single-joint exercises, which are commonly employed in early-stage rehabilitation and athletic conditioning programs. **Results**: Application of the model to single-joint knee extension exercises demonstrated that the anterior cruciate ligament (ACL)-loading shear tibiofemoral force can be entirely eliminated throughout the full range of knee motion, without increasing either the tibiofemoral compressive force or the posterior cruciate ligament (PCL)-loading shear component, while preserving the desired peak and profile of the resistance torque. **Conclusion**: The proposed analytical framework enables a comprehensive understanding of how to regulate resistance parameters through advanced electromechanical technologies to minimize joint stress during single-joint strengthening exercises. Precise control of joint reaction forces during exercise is critical for the design of therapeutic and safety-enhanced training protocols.

## 1. Introduction

The force produced by a muscle depends on its physiological cross-sectional area, the instantaneous length of its fibers, and their rate of length change, as well as on the degree of activation, which is controlled by neural commands through the types, numbers, and firing rates of the recruited motor units [1]. A muscle exerts force on bone segments at the tendon-bone interfaces and mobilizes the intervening joints, unless these movements are counteracted by forces from other muscles, joint structures, or external resistances. The articulating bone segments rotate around instantaneous joint axes of rotation, which are never perfectly fixed in space as joint motion progresses [2]. The contribution of the muscle force $\vec{F}$ to joint motion over time is determined by the axial torque (or axial moment) of $\vec{F}$ about the instantaneous joint axis [3]. This torque equals the product of the muscle force moment arm $a_{F}$ (the minimum distance between the muscle force line of action and the joint instantaneous axis) and the magnitude of the muscle force component normal to the joint axis [4]. The maximum moment that a muscle can exert about a joint axis varies across the joint range of motion, reflecting changes in both maximum muscle force (the force of the fully activated muscle) and moment arm [5], and ultimately depends on the instantaneous values of the angles and angular velocities of the joints crossed by the muscle. These quantities determine the moment arm, and the instantaneous length and shortening velocity of the muscle fibers.

During a single-joint exercise, joint kinematics is determined by the net moment about the joint axis generated by the external forces acting on the exercising segment or limb. The moments of agonist muscle forces, the opposing moments of antagonistic muscle forces, and the moments arising from the reactive forces of passive joint structures (bone-to-bone contact forces, ligament tension, etc.) collectively constitute the resultant joint moment, $\tau_{j}$ [6]. In maximal single-joint efforts, the resultant joint moment, $\tau_{j}^{max}$, inherits its dependence on joint angle θ and joint angular velocity $\dot{\theta }$ from the moments of the muscle forces. The set of curves describing the dependence of $\tau_{j}^{max}$ on θ at constant $\dot{\theta }$ values are referred to as isokinetic curves, or strength curves, for the joint movement [7]. These curves typically show an ascending–descending trend, whose shape depends on both the joint and the type of movement [8]. However, exceptions exist; for example, knee flexion isokinetic curves display a monotonically descending pattern [7].

Isokinetic curves at different angular velocities can be measured experimentally using an isokinetic dynamometer. This device maintains angular velocity nearly constant at a preselected value by continuously equalizing the external resistance torque to the joint torque throughout the movement [9]. The isokinetic dynamometer is a key tool for strength assessment and can also be used to perform isokinetic exercise in strength training and rehabilitation programs [10,11].

Most single-joint strength training machines provide a resistance torque profile designed to approximate the population-average strength curve obtained by isokinetic dynamometry [12]. This is commonly achieved by incorporating a properly shaped cam (asymmetrical pulley) into the equipment mechanics [12,13]. The peak resistance torque can be selected by the user without changing the relative torque profile, i.e., the percentage variation in the torque across the range of motion (ROM) [13]. As a result, muscle activation remains nearly constant if the exercise is performed at a controlled velocity, and the terms “isotonic equipment” and “isotonic exercise” are often used in this context, albeit improperly. Due to the lack of velocity control and torque feedback regulation, these machines are relatively inexpensive compared to isokinetic devices. However, they cannot accommodate intersubject differences in strength curves.

Both isokinetic and isotonic devices present important limitations in athletic training. The isokinetic device enforces a constant angular velocity, which seldom reflects real-life activities and sports movements [14]. In isotonic equipment, inertial effects associated with the accelerations of moving masses (weight stacks and levers) can significantly distort the nominal torque profile generated by the cam, especially during explosive movements with low and medium resistances [15]. Cam design cannot adequately compensate for inertial effects, because these forces depend on the acceleration pattern of the exercise, which cannot be predicted in advance.

Importantly, neither isokinetic nor isotonic devices are designed to control or minimize the loads acting on joint structures, including articular surfaces, ligaments, and other periarticular tissues. Understanding and regulating the joint reaction forces that arise during exercise is fundamental for designing appropriate rehabilitation and conditioning programs and for preventing injuries [16,17]. Therefore, it is crucial to devise a new generation of safe and effective strength machines that optimize both muscular and joint aspects of exercise, overcoming the limitations of existing equipment.

Joint reaction forces are determined by muscle forces, external resistance, and instantaneous kinematic parameters [18]. With modern electromechanical technologies, resistance magnitude, direction, and application point can be regulated during the exercise, either in a preprogrammed mode or adaptively in response to real-time kinematic data. Such features have recently been integrated into strength machines to replicate functional, sport-specific explosive movements [19]. However, their potential to precisely control the mechanical load on specific joint structures has yet to be explored. In particular, existing systems regulate resistance primarily to reproduce kinematic or torque targets rather than to analytically control joint reaction forces. To the best of our knowledge, no previous analytical formulation has provided a closed-form relationship that simultaneously links joint reaction force components to instantaneous joint kinematics and to independently adjustable resistance parameters (magnitude, direction, and point of application) under an arbitrarily prescribed resistance–torque constraint.

The aim of this study is to establish a framework of fundamental biomechanical equations to exploit these next-generation devices for the control and minimization of joint reaction forces during strengthening exercises. The focus is on analytically determined single-joint movements, which are commonly used in early rehabilitation and athletic training programs. The analytical approach reveals the functional relationships between joint reaction forces and their determining parameters, providing a theoretical basis for regulating these parameters to reduce joint stress. The proposed analytical framework is subsequently examined through its application to the leg extension exercise, which serves as a representative case study for analytically tractable single-joint movements. This work represents a foundational step toward the development of intelligent strength machines that assist trainers and therapists in preventing injuries and optimizing rehabilitation strategies.

## 2. Materials and Methods

**Figure 1:**
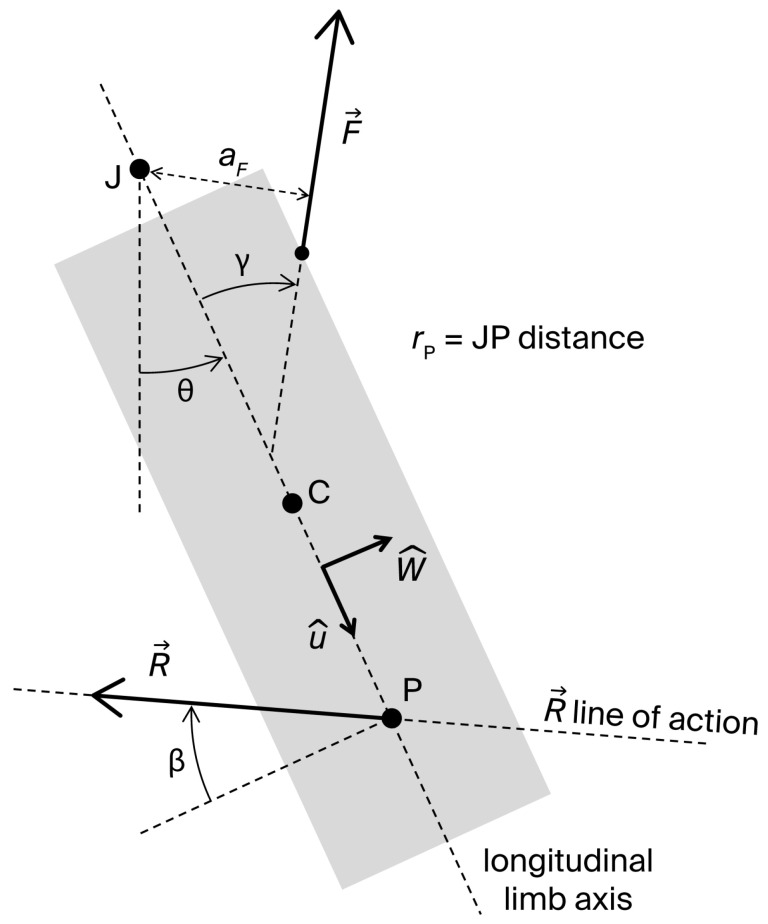
Two-dimensional sketch of the moving segment in a single-joint exercise with its fundamental elements: longitudinal axis; center of mass (C); joint center of rotation (J); joint angle θ, defined as the angle between the longitudinal limb axis and a reference direction; muscle force $\vec{F}$; traction angle (γ) and moment arm ($a_{F}$) of $\vec{F}$; external resistance, equivalent to a vector $\vec{R}$ applied at point P of the longitudinal axis; angle (β) that defines the line of action of $\vec{R}$; and JP distance ($r_{P})$.

Figure 1 displays a two-dimensional sketch of a single-joint strength exercise and its fundamental elements: the longitudinal axis $\left(a_{long}\right)$, center of mass (C), and center of rotation (J) of the exercising limb, considered as a rigid body; the joint angle θ (defined as the angle between the longitudinal limb axis and a reference direction); a muscle force $\vec{F}$ applied at the insertion point I; and the external resistance $\vec{R}$ applied at point P. Multiple external resistances may act on the system in the plane of movement, including the limb weight $m\vec{g}$ applied at C. Nevertheless, a planar system of forces can always be reduced to a single force $\vec{R}$ applied at a point P. Furthermore, since $\vec{R}$ can be freely displaced along its line of action, P can be located on $a_{long}$ without loss of generality. We assume that: (1) J is fixed; (2) C and J lie on $a_{long}$; and (3) the effects of muscle forces other than the dominant agonist force $\vec{F}$ can be neglected. The moving limb joint is also subject to the passive joint reaction forces arising from contacts between articulating surfaces and tension in capsuloligamentous tissues. The resultant of these forces is equivalent to a vector $\vec{\phi }$ applied at a point within the joint.

Under the given assumptions, the dynamic moment equation of the system about the joint axis is:

(1) $$I\ddot{\theta }=a_{F}F-Rr_{P}\cos\beta +\tau_{\phi }$$

where $a_{F}$ is the moment arm of $\vec{F}$, I is the moment of inertia of the system about J, $\ddot{\theta }$ is the joint angular acceleration, $r_{P}$ is the JP distance, β is the angle defining the direction of $\vec{R}$ (as shown in Figure 1; −90°<β<90°, and β=0 corresponds to a resistance acting perpendicularly on $a_{long}$), and $\tau_{\phi }$ is the axial moment generated by $\vec{\phi }$ about J. Although $\tau_{\phi }$ is unknown, it can be considered negligible except at the extremes of the ROM ([20], pp. 68–73). Typically, $\tau_{\phi }$ reaches considerable values at end-range to limit further joint movement. Equation (1) can be rearranged to determine the magnitude F of the muscle force generating the movement:

(2) $$F=\frac{1}{a_{F}}\left(I\ddot{\theta }+Rr_{P}\cos\beta -\tau_{\phi }\right)$$

The joint reaction force $\vec{\phi }$ can be derived from the dynamic force equation applied to the moving limb:

(3) $$m{\vec{a}}_{C}=\vec{F}+\vec{\phi }+\vec{R}$$

where the acceleration of C, ${\vec{a}}_{C}$, given the assumed rigid body scheme of the limb, can be expressed as:

(4) $${\vec{a}}_{C}=r_{C}\ddot{\theta }\hat{w}-r_{C}{\dot{\theta }}^{2}\hat{u}$$

Here, $r_{C}$ is the JC distance, $\dot{\theta }$ is the joint angular velocity, $\hat{u}$ is the unit vector of $a_{long}$, and $\hat{w}$ is the unit vector normal to $\hat{u}$, pointing in the direction of movement (Figure 1). Projecting Equations (3) and (4) along $\hat{w}$ and $\hat{u}$, and denoting by γ the traction angle of $\vec{F}$ (i.e., the angle of $\vec{F}$ relative to $-\hat{u}$) one obtains the scalar equations:

(5) $$mr_{C}\ddot{\theta }=F\sin\gamma -R\cos\beta \;+\phi_{shear}$$

(6) $$-mr_{C}{\dot{\theta }}^{2}=-F\cos\gamma -R\sin\beta +\phi_{axial}$$

These equations determine the shear and axial components, $\phi_{shear}$ and $\phi_{axial}$, of $\vec{\phi }$:

(7) $$\phi_{shear}=mr_{C}\ddot{\theta }-F\sin\gamma +R\cos\beta$$

(8) $$\phi_{axial}=-mr_{C}{\dot{\theta }}^{2}+F\cos\gamma +R\sin\beta$$

Positive values of $\phi_{axial}$ correspond to a compressive axial force on the joint surfaces; negative values indicate traction. Positive $\phi_{shear}$ values correspond to an anterior ($\hat{w}$-oriented) force exerted on the moving segment by the adjacent distal segment—i.e., a posterior ($-\hat{w}$-oriented) force exerted by the moving segment on the distal fixed one. Conversely, negative $\phi_{shear}$ values correspond to a posterior force exerted on the moving segment by the adjacent distal segment—i.e., an anterior force exerted by the moving segment on the distal fixed one. For example, in a single-joint knee extension exercise, a positive $\phi_{shear}$ represents an anterior force exerted by the femur on the tibia, or a posterior force by the tibia on the femur, which induces a load on the posterior cruciate ligament (PCL). A negative $\phi_{shear}$ corresponds to the reverse condition, involving a load on the anterior cruciate ligament (ACL).

Substituting Equation (2) into Equations (7) and (8) yields:

(9) $$\phi_{shear}=\left(mr_{C}-\frac{\sin\gamma }{a_{F}}I\right)\ddot{\theta }+R\cos\beta \left(1-\frac{\sin\gamma }{a_{F}}r_{P}\right)+\frac{\sin\gamma }{a_{F}}\tau_{\phi }$$

(10) $$\phi_{axial}=-mr_{C}{\dot{\theta }}^{2}+\frac{\cos\gamma }{a_{F}}I\ddot{\theta }+R\left(\sin\beta +\frac{\cos\gamma }{a_{F}}r_{P}\cos\beta \right)-\frac{\cos\gamma }{a_{F}}\tau_{\phi }$$

Given that $\tau_{\phi }\sim 0$ (except near the ROM extremes), Equations (2), (9) and (10) express *F*, $\phi_{shear}$, and $\phi_{axial}$ in terms of measurable parameters related to the muscle architecture ($a_{F}(\theta )$ and γ(θ)), limb inertia (m, $r_{C}$, and I), resistance (R, $r_{P}$, and β), and movement kinematics ($\dot{\theta }$ and $\ddot{\theta }$). Notably, these equations highlight how resistance parameters can be adaptively managed to control $\phi_{shear}$ and $\phi_{axial}$ during the exercise. Indeed, modern electromechanical technologies allow real-time variation in resistance magnitude (R), direction (β), and point of application ($r_{P}$), either via pre-programmed profiles (e.g., functions of θ), or adaptively in response to instantaneous values of $\dot{\theta }$ and $\ddot{\theta }$. However, the regulation of $\phi_{shear}$ and $\phi_{axial}$ must not compromise the effectiveness of the strengthening stimulus, which is determined by the preselected optimal/desired profile $\tau_{R}^{opt}\left(\theta \right)$ of resistance torque $Rr_{P}\cos\beta$, which, together with $\ddot{\theta }$, determines the muscle force (see Equation (2)). Thus, R, $r_{P}$, and β should be varied under the constraint:

(11) $$Rr_{P}\cos\beta =\tau_{R}^{opt}\left(\theta \right)\;$$

Analytically, the problem can be formulated as follows: given the functions $a_{F}(\theta )$ and γ(θ), and the values of m, $r_{C}$, and I, the adaptive regulation of R, β, and $r_{P}$ in response to the instantaneous values of θ, $\dot{\theta }$, and $\ddot{\theta }$ is used to minimize or control $\phi_{shear}$ (Equation (9)) and $\phi_{axial}$ (Equation (10)), without altering the designed variable resistance torque profile $Rr_{P}\cos\beta$ (Equation (11)). Of course, exercisers may also intentionally modulate $\dot{\theta }$ and $\ddot{\theta }$ to influence $\phi_{shear}$ and $\phi_{axial}$, for example, by moving the limb quasi-statically ($\dot{\theta }=\ddot{\theta }\approx 0$), or by minimizing acceleration peaks ($\ddot{\theta }\approx 0$). Nevertheless, when repetitions are performed continuously, even at low velocity, peak accelerations at movement inversion points may become considerable.

In the following, we specifically analyze an open kinetic chain (OKC), single-joint knee-extension exercise, in which the dominant muscular action is provided by the quadriceps. In this case, $\vec{F}$ represents the patellar tendon force; $a_{F}$ and γ denote the moment arm and the traction angle of the patellar tendon, respectively; and $\phi_{shear}$ and $\phi_{axial}$ are the shear and axial components of the tibiofemoral reaction force. The dependences of γ and $a_{F}$ on the knee angle θ were taken from the data of Herzog and Read [21]. The inertial parameters were derived from the data reported by Winter ([20], p. 83) and Enoka ([22], p. 73), assuming a male subject 175 cm tall and weighing 75 kg. By convention, θ=0 corresponds to 90° of knee flexion, and θ=90° to full knee extension. The knee flexion angle $\theta_{\mathrm{flex}}$ is therefore given by $\theta_{\mathrm{flex}}=90{}^{\circ}-\theta$. The exercise is performed within the range $0{}^{\circ}\leq \theta_{\mathrm{flex}}\leq 120{}^{\circ}$.

All computations were performed analytically, and the graphs were generated using Origin (OriginLab Corporation, Northampton, MA, USA).

Regarding the quantification of passive joint torque at full knee extension ($\tau_{\phi }$), experimental studies report values that fall within a broadly consistent range despite differences in modeling assumptions, subject variability, and the exponential increase in passive tension characteristic of terminal extension. Silder et al. [23] reported a value of approximately 8 Nm, and Mansour and Audu [24] values of 10–15 Nm. Riener and Edrich [25] measured a baseline passive moment of about 2.5 Nm, with an additional contribution of roughly 9 Nm probably attributable to the joint capsule. Taken together, these findings indicate that a value on the order of 10 Nm represents a reasonable and physiologically grounded estimate for modeling $\tau_{\phi }$. Importantly, within the present analytical formulation, the influence of $\tau_{\phi }$ on the axial and shear components of the tibiofemoral joint reaction force is modulated only by the architectural parameters of the extensor mechanism ($a_{F}$ and γ; see Equations (9) and (10)).

## 3. Results

### 3.1. Axial Component of the Joint Reaction Force

According to Equation (10), the angular velocity-dependent term $-mr_{C}{\dot{\theta }}^{2}$ always generates a traction force ($\phi_{axial}<0$) throughout the ROM. In a leg extension exercise with light resistance, peak angular velocity of approximately 500°/s can be reached [26], resulting in a traction force of nearly 90 N.

The effect of the angular acceleration-dependent term $I\left(\cos\gamma /a_{F}\right)\ddot{\theta }$ depends on the sign of cosγ. For cosγ>0, this term produces a compressive force ($\phi_{axial}>0$) at the beginning of the movement ($\ddot{\theta }>0$), and a traction force ($\phi_{axial}<0$) in the final phase ($\ddot{\theta }<0$). This pattern characterizes the leg extension exercise, in which the traction angle γ of the patellar tendon force increases from approximately −10° to 20° as the knee extends from a flexion angle of 120° [21]. In the case cosγ<0, the opposite behavior occurs. In a low-resistance leg extension lasting approximately 0.5 s and assuming a parabolic velocity profile, angular acceleration can reach values as high as 4000°/s^2^ as movement begins, generating a compressive force of about 550 N.

The axial component $\phi_{axial}$ can also be modulated by R, $r_{P}$, and β through the factor $R\left(\sin\beta +\frac{\cos\gamma }{a_{F}}r_{P}\cos\beta \right)$. Notably, in quasi-static leg extension exercises ($\dot{\theta }=\ddot{\theta }\approx 0$), $\phi_{axial}$ can, in principle, be reduced to zero—except at the extreme points of the ROM, where $\tau_{\phi }$ cannot be neglected—when $\left(\sin\beta +\frac{\cos\gamma }{a_{F}}r_{P}\cos\beta \right)=0$, that is when $r_{P}$ and β are continuously adjusted throughout the movement to meet the condition:

(12) $$\frac{\tan\beta }{r_{P}}=-\frac{\cos\gamma }{a_{F}}$$

**Figure 2:**
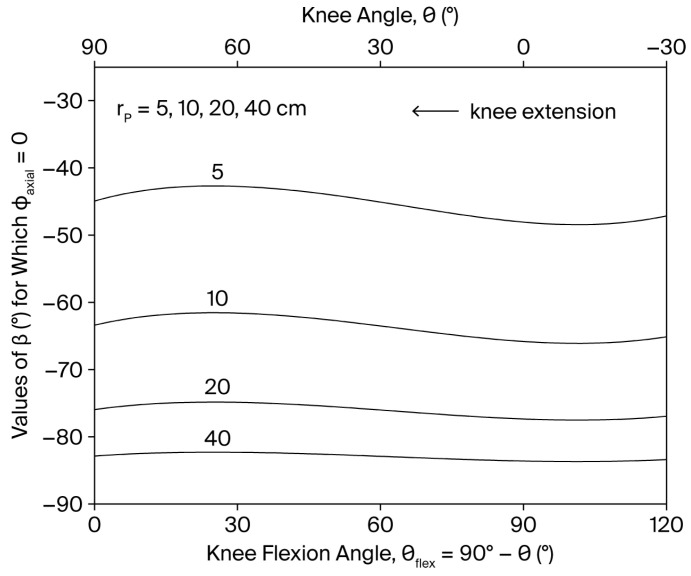
Dependence of the values of β that reduces $\phi_{axial}$ to zero on the knee angle θ and knee flexion angle $\theta_{\mathrm{flex}}$ ($\theta_{\mathrm{flex}}=90{}^{\circ}-\theta$) for different values of $r_{P}$ ($r_{P}=5,\;10,\;20,\;40\;cm$) during a single-joint, OKC knee extension exercise. β defines the line of action of the resistance $\vec{R}$ (see Figure 1).

Since the right-hand side is always negative, β should be varied in range −90°<β<0 (in leg extension exercise −90°<β<90° for the resistance to provide opposition to knee extension). As shown in Figure 2, when $r_{P}$ is kept constant during the movement, the values of β required to reduce $\phi_{axial}$ to zero are approximately −83° when the resistance is applied distally on the shank ($r_{P}=0.4$ m), a condition that may require a complex resistance-setting strategy involving multiple force components (see Appendix A). However, as the point of application of the resistance is gradually shifted proximally, the condition $\phi_{axial}=0$ is satisfied for progressively higher values of β, reaching about −45° in the extreme case $r_{P}=0.05$ m. To preserve the desired variable resistance profile $\tau_{R}^{opt}\left(\theta \right)$, R must be varied simultaneously across the ROM so that $Rr_{P}\cos\beta =\tau_{R}^{opt}\left(\theta \right)$.

At the end of the range of motion (where $\tau_{\phi }<0$), the term $-\left(\cos\gamma /a_{F}\right)\tau_{\phi }$ becomes significantly positive and contributes to a compressive joint force. While negligible during most of the movement, this effect becomes relevant at end range, with a magnitude of about 200 N.

### 3.2. Shear Component of the Joint Reaction Force

**Figure 3:**
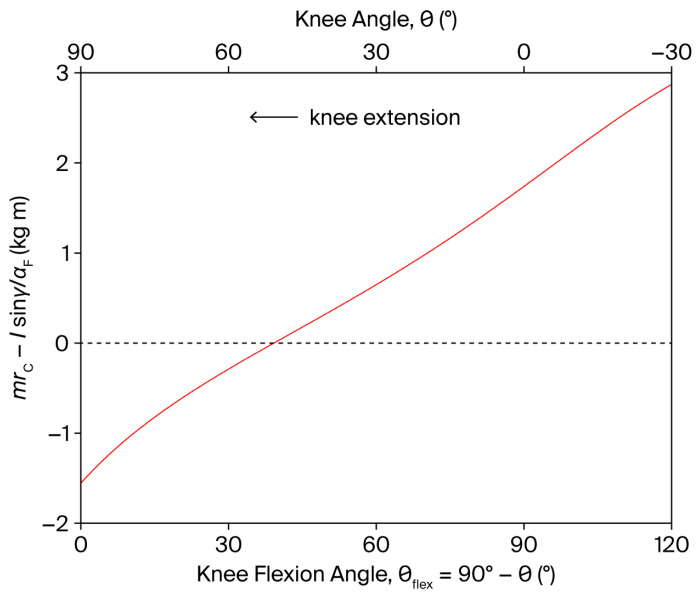
Dependence of $\left(mr_{C}-I\sin\gamma /a_{F}\right)\ddot{\theta }$ on the knee angle θ and knee flexion angle $\theta_{\mathrm{flex}}$ ($\theta_{\mathrm{flex}}=90{}^{\circ}-\theta$), during a single-joint, OKC knee extension exercise (red line). The shear component $\phi_{shear}$ of the tibiofemoral joint reaction force is independent of the angular velocity $\dot{\theta }$ and is modulated by the angular acceleration $\ddot{\theta }$ through the factor $\left(mr_{C}-I\sin\gamma /a_{F}\right)\ddot{\theta }$ (see Equation (9)). The dashed line represents the zero level of the dependent variable.

The shear component $\phi_{shear}$ is independent of the angular velocity $\dot{\theta }$ and is modulated by the angular acceleration $\ddot{\theta }$ through the factor $\left(mr_{C}-I\sin\gamma /a_{F}\right)\ddot{\theta }$ (Equation (9)). In a leg extension exercise, this factor is positive both at the beginning ($\ddot{\theta }>0$) and at the end ($\ddot{\theta }<0$) of the movement, as the term in parentheses changes sign, as shown in Figure 3. Consequently, a PCL-loading shear force ($\phi_{shear}>0$) is induced by $\ddot{\theta }$ in both movement phases. For instance, an angular acceleration of 4000°/s^2^ at movement onset can generate a shear force of approximately 24 N.

The resistance parameters R, $r_{P}$, and β can influence $\phi_{shear}$ through the factor $R\cos\beta \left(1-r_{P}\sin\gamma /a_{F}\right)$. Notably, when $\ddot{\theta }\approx 0$, $\phi_{shear}$ can always be maintained positive, i.e., of PCL-loading type—except at the extremes of the ROM where $\tau_{\phi }$ cannot be neglected—when the point of application of the resistance is displaced along the anterior aspect of the tibia such that:

(13) $$r_{P}(\theta )\leq \frac{a_{F}(\theta )}{\sin\gamma (\theta )}$$

**Figure 4:**
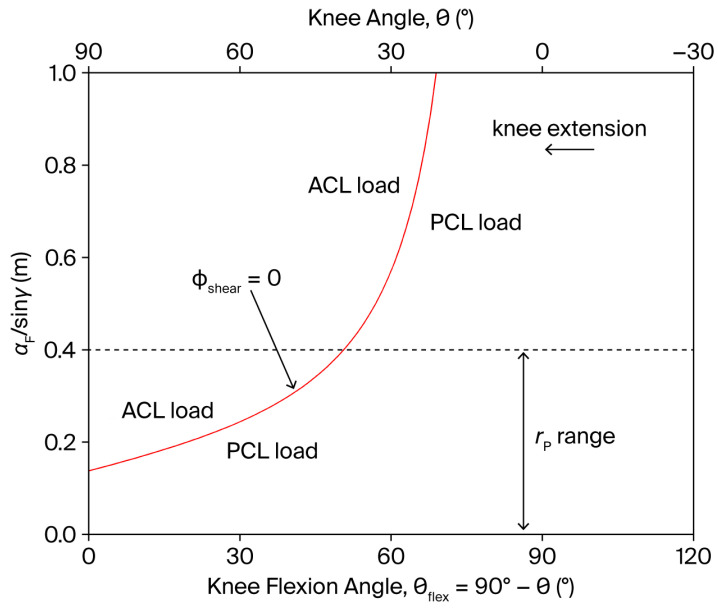
Dependence of the factor $a_{F}/\sin\gamma$ on the knee angle θ and knee flexion angle $\theta_{\mathrm{flex}}$ ($\theta_{\mathrm{flex}}=90{}^{\circ}-\theta$), during a single-joint, OKC knee extension exercise (red line). The shear component $\phi_{shear}$ of the tibiofemoral joint reaction force can ideally be reduced to zero when $r_{P}=a_{F}/\sin\gamma$ (see Figure 1 for the definition of $r_{P}$, $a_{F}$, and γ). The dashed line represents the approximate upper anatomical limit of $r_{P}$.

In particular, $\phi_{shear}=0$ when $r_{P}=a_{F}/\sin\gamma$. However, to preserve the desired resistance torque profile $\tau_{R}^{opt}\left(\theta \right)$ (Equation (11)), R and/or β must be simultaneously regulated in coordination with $r_{P}$ throughout the movement. The functional dependence of $a_{F}/\sin\gamma$ on θ is displayed in Figure 4. Since $r_{P}$ can generally be set in the range 5–40 cm (the tibial length is approximately 43 cm), a PCL-loading shear force ($\phi_{shear}>0$) always persists for θ>50°, regardless of the resistance point position. Conversely, for θ≤50°, the point of application of the resistance can, in principle, be displaced towards the knee during the movement to reduce $\phi_{shear}$ to zero in the whole 0°≤θ≤50° subrange of motion.

At the end of the range of motion (where $\tau_{\phi }<0$), the term $\left(\sin\gamma /a_{F}\right)\tau_{\phi }$, being negative, contributes to an ACL-loading shear force ($\phi_{shear}<0$) of about 57 N.

## 4. Discussion

The present study formally derives the analytical relationship between joint reaction forces—specifically, shear and axial components—and both the kinematic descriptors of joint motion (joint angle, angular velocity, and angular acceleration) and the externally imposed resistance conditions (magnitude, direction, and application point), within the context of a single-joint strengthening task (Equations (9) and (10)). The integration of contemporary electromechanical actuation systems enables continuous modulation of these resistance parameters throughout the execution of the movement, either through predefined control algorithms or via adaptive schemes responsive to instantaneous kinematic states. This dynamic controllability facilitates the accurate targeting of mechanical loads on specific articular structures, which is pivotal for mitigating injury risk and for the systematic optimization of rehabilitative interventions, although experimental validation will be required to confirm these theoretical predictions under real training and rehabilitation conditions.

Importantly, the proposed framework should not be interpreted as solely aiming to minimize joint reaction forces. While limiting excessive loads may be desirable in early rehabilitation or injury-prevention contexts, physiological loading is essential for maintaining cartilage integrity, ligament strength, and overall joint health through mechanobiological adaptation. The analytical relationships derived here also provide insight into how resistance parameters may be adjusted to maintain forces within prescribed ranges aligned with specific therapeutic or training objectives, including progressive loading strategies that support tissue adaptation or remodeling. To this end, Appendix B reports literature-based ACL loading ranges and illustrates how the calculated shear force relates to physiologically meaningful levels.

A practical challenge concerns the behavior of the system under highly dynamic conditions, particularly when movements involve non-smooth velocity profiles or large angular accelerations. Because angular acceleration is explicitly included in the governing equations, the proposed analytical framework remains valid for arbitrary movement dynamics. Although acceleration-dependent terms may increase joint reaction forces, they do not alter the analytical relationships linking kinematics, resistance parameters, and joint loading. Accordingly, this limitation does not arise from the model itself but rather from the performance requirements imposed on the experimental or training equipment. In practice, accurate implementation requires instrumentation capable of rapid response and precise control. As detailed in the section “Actuator and Sensor Specifics” of Appendix A, modern adaptive electromechanical resistance systems equipped with high-resolution sensors, sufficient actuator power, and fast control bandwidth can compensate in real time for acceleration-dependent effects on joint loading and resistance torque.

The subject considered in the illustrative example does not represent a specific reference individual but serves solely to provide a numerical instantiation of the analytical relationships. Because anthropometric and inertial parameters enter the formulation explicitly, the model is inherently scalable to different body sizes and segment properties. Subject-specific characteristics can be directly incorporated by substituting the corresponding anthropometric data (e.g., through established anthropometric scaling procedures based on published segmental datasets ([20], p. 83), [22] without modifying the structure of the governing equations.

From a mechanical standpoint, the influence of anthropometric variability on joint reaction forces follows directly from the explicit presence of segment masses, centers of mass, and moments of inertia in the dynamic equations. Inertial contributions scale proportionally with segment mass and angular acceleration, whereas the geometric relationships governing the optimal resistance configuration remain unchanged. Accordingly, the sensitivity of joint reaction forces to angular acceleration is preserved across subjects, while only their absolute magnitude scales with anthropometric parameters. Consequently, differences among subjects (e.g., elderly individuals versus trained athletes) primarily affect the magnitude of the computed forces rather than the structure of the resistance-setting strategy required to control joint loading.

As an illustrative example, according to the anthropometric data of de Leva ([22], p. 73), the combined mass of the leg–foot segment represents on average approximately 6% of total body mass in both males and females. A 10% variation in body mass therefore produces a proportional change in the associated inertial terms; in absolute terms, however, this corresponds to only about 0.6% of total body mass. These terms become mechanically relevant only in movements involving high angular velocities or accelerations, whereas they remain negligible during controlled exercise conditions typical of single-joint exercises. A similar proportional scaling applies to the segment moment of inertia. This example highlights that anthropometric variability is naturally accommodated through direct parameter substitution, without altering the analytical structure of the model.

### 4.1. Axial Joint Reaction Force

Compressive forces play a fundamental role in joint stability by resisting forces that tend to reduce articular congruence. Specifically, the presence of a compressive load attenuates shear-induced sliding translation between articular surfaces, thereby diminishing the tensile demand on capsuloligamentous structures that typically counteract such displacement [27]. Nonetheless, when these compressive loads exceed physiological thresholds, they may impose deleterious mechanical stress on periarticular connective tissues interposed between opposing joint surfaces—such as articular cartilage, intervertebral disks, and menisci—thus increasing the risk of structural degeneration or failure [28].

In the context of knee extension exercises, the application of a traction force aligned with the tibial axis reliably induces separation of the tibial plateaus from the femoral condyles. This mechanically induced distraction is often exploited in rehabilitative protocols to reduce intra-articular loading, relieve pain, or promote capsular elongation [29]. Notably, $\phi_{axial}$ can, in theory, be entirely eliminated, without altering the prescribed resistance torque profile, by defining the functions $r_{P}(\theta )$ and β(θ) such that the condition expressed in Equation (12) is satisfied. Conversely, the application of an external axial compression has the potential to limit the anterior tibial translation induced by the quadriceps force during the final phase of knee extension. Within such frameworks, the axial component of the tibiofemoral joint reaction force ($\phi_{axial}$) can be directly modulated according to Equation (9).

### 4.2. Shear Joint Reaction Force

The shear component of the joint reaction force ($\phi_{shear}$) typically generates tensile strain on capsuloligamentous structures. In the knee joint, anterior and posterior tibial translations are primarily constrained by the ACL and PCL, respectively [30]. Consequently, minimizing $\phi_{shear}$—ideally reducing it to zero—is essential when the goal is to limit capsuloligamentous loading during strengthening exercises. Consistent with the trends predicted by the present analytical formulation, evidence from in vivo, in vitro, and numerical simulation studies shows that, during open kinetic chain knee extension with resistance applied distally on the lower leg, an anteriorly directed tibial shear force develops in the final portion of the extension range (approximately the last 40–50°), corresponding to increased ACL loading (references are collectively collected in [31]). It has also been experimentally demonstrated that a more proximal placement of the resistance pad reduces this anterior shear component [32]. These independent findings support the mechanical plausibility of the relationships described by the present model. In single-joint OKC knee extension exercises, accordingly, the ACL-loading component of $\phi_{shear}$ can be entirely eliminated by avoiding significant accelerations ($\ddot{\theta }\approx 0$) and forced terminal extension ($\tau_{\phi }\approx 0$), provided that the resistance application point (determined by $r_{P}$) is shifted proximally towards the knee during the final 50° of extension, as described in Equation (13) (see Figure 4). When accompanied by concurrent modulation of R, this adjustment enables the suppression of ACL-loading shear force without altering the resistance torque profile (and the level of muscle activation, under conditions where $\ddot{\theta }$ and $\tau_{\phi }$ remain negligible), nor affecting the compressive tibiofemoral force and the PCL-loading component of $\phi_{shear}$, which is typically engaged at knee flexion angles exceeding 50°.

This principle was originally theorized by the author in a previous work [13], using a conceptual model of a cam-equipped leg extension machine integrated with a movable resistance pad. However, with this equipment setting, the displacement of the resistance pad along the anterior surface of the tibia is predetermined and cannot compensate for the ACL-loading component of $\phi_{shear}$ that arises from knee angular accelerations, as described in Equation (9). As a result, this strategy remains effective only under quasi-static conditions. By contrast, modern electromechanical systems enable real-time adaptive control of resistance parameters, thereby allowing dynamic compensation for the $\ddot{\theta }$-dependent component of $\phi_{shear}$. This technological advancement extends the protective and functional benefits of the strategy to dynamic and high-velocity exercises. The analytical foundation for this form of adaptive regulation is provided by Equation (9), which incorporates the influence of both angular acceleration and resistance parameters into the computation of $\phi_{shear}$. Interestingly, in such exercises, the target resistance torque profile should be modulated adaptively to account for the inertial torque $I\ddot{\theta }$ associated with the angular acceleration of the shank-foot system (Equation (2)). Specifically, the ideal quasi-static torque profile should be decreased by $I\ddot{\theta }$ during the initial acceleration phase of the exercise ($\ddot{\theta }>0$), and increased by $I\left|\ddot{\theta }\right|$ during the final deceleration phase ($\ddot{\theta }<0$). This can be achieved through a corresponding modulation of the resistance magnitude R, without interfering with the preprogrammed displacement of the resistance application point—governed by Equation (13)—which is designed to suppress ACL loading. In addition, cam-equipped leg extension machines provide a population-average strength curve derived from isokinetic dynamometry data, whereas adaptive machines are capable of recording and subsequently reproducing the individual user’s strength profile. This profile may differ substantially from the population-average curve, particularly in the presence of neuromusculoskeletal impairments or pathological conditions.

### 4.3. Limitations and Future Developments

This study focused exclusively on open kinetic chain (OKC), single-joint exercises characterized by the dominance of a primary agonist muscle. Contributions from synergistic and antagonistic muscles were assumed to be negligible. This assumption is generally valid in movements such as OKC knee extension or elbow extension, which are predominantly driven by the quadriceps femoris and triceps brachii, respectively. In these contexts, antagonist muscle activity is typically minimal, except near the terminal range of motion, where reflexive co-contractions may occur to protect joint structures. For instance, during dynamic OKC knee extension, hamstring activation near full extension can generate a compressive, posteriorly directed force on the tibia, which partially unloads the ACL [32,33,34,35,36].

The magnitude of hamstring co-activation during controlled OKC movements is generally minimal relative to the dominant quadriceps force, remains largely confined to the terminal phase of knee extension, and may exhibit substantial inter-individual variability influenced by neuromuscular coordination, training status, age, and exercise familiarity ([22], p. 353). Accordingly, incorporating this contribution into a deterministic planar mechanical model would require subject-specific activation patterns that fall beyond the scope of the present analysis. Importantly, neglecting hamstring co-activation results in a conservative estimation of anterior tibial shear and ACL loading. Specifically, under the condition $r_{P}=a_{F}/sin\gamma$, for which $\phi_{\mathrm{shear}}=0$ in the absence of hamstring co-contraction (Equation (13)), additional hamstring activity would introduce a posterior shear component, thereby slightly increasing PCL loading. However, given both the limited magnitude of hamstring co-activation in OKC knee extension and the greater structural robustness of the PCL relative to the ACL, the resulting increase in posterior shear is biomechanically negligible under the loading conditions considered.

Additional simplifying assumptions concern the representation of the limb as a rigid segment and the adoption of a fixed joint center. In the leg extension exercise, the migration of the instantaneous knee center of rotation during extension is negligibly small relative to the distance between the joint center J and the resistance application point P; accordingly, its influence on the computed axial and shear components of the tibiofemoral joint reaction force has previously been shown to be negligible [3]. Importantly, although this migration has negligible influence on the calculated joint reaction forces, it must not be mechanically constrained by the equipment, since restricting physiological joint center translation may substantially alter arthrokinematics and thereby significantly affect joint loading. Likewise, representing the limb as a rigid segment does not allow direct estimation of how the net tibiofemoral shear force is distributed among individual periarticular soft-tissue structures and their deformation. However, experimental evidence indicates that the anterior cruciate ligament provides approximately 86% of the total restraining force to anterior tibial translation [18], which represents the mechanically relevant quantity for the present analysis.

From a modeling standpoint, the present analytical formulation is based on a two-dimensional representation of joint mechanics and therefore does not capture the full complexity of three-dimensional joint behavior. However, this simplification is appropriate within the specific context of monoarticular, mono-planar exercises, where joint alignment can be mechanically constrained and out-of-plane deviations are minimized. For example, during knee extension performed on a leg extension machine, the thigh and popliteal region are stabilized against the seat, and the resistance roller applies force in a controlled sagittal-plane direction. Under these conditions, varus–valgus deviations and tibial internal–external rotations—mechanisms that are critical in ACL injury during complex dynamic tasks—are typically negligible.

More complex motor tasks—such as multi-joint exercises involving the coordinated recruitment of multiple muscle groups—fall outside the scope of the present analytical framework. In such cases, the system becomes mechanically indeterminate, requiring the use of numerical simulations and assumption-based optimization methods to estimate individual muscle forces [37,38,39,40]. As a consequence, the relationship between kinematic and resistance parameters and joint reaction forces can no longer be captured by a general closed-form analytical expression, but must instead be continuously recomputed as the movement progresses. This constraint reduces the immediate interpretability of the model and substantially limits both preprogrammed and real-time adjustment of resistance parameters based on instantaneous kinematic values (position, velocity, and acceleration)—a critical requirement for the precise control of joint loading during therapeutic exercise and for safety-enhanced exercise conditions intended to limit excessive joint stress and functional overuse.

Nevertheless, these challenges do not represent fundamental barriers, but rather indicate directions for future research. In particular, the integration of a general numerical biomechanical modeling framework with real-time physiological inputs, such as electromyographic signals acquired during exercise, may enable time-varying estimation of muscle forces and joint reaction loads during multi-joint movements. Coupled with advanced adaptive electromechanical resistance technologies, such approaches could enable continuous adjustment of resistance magnitude, direction, and point of application during exercise to minimize joint stress, thereby extending the principles established in the present work to more complex motor tasks. Further research is warranted to explore these developments and to assess their feasibility, robustness, and clinical relevance.

## 5. Conclusions

This study provides an analytical foundation for the control of joint reaction forces in single-joint strengthening exercises through modulation of resistance parameters. By formalizing the relationships between kinematic and resistance inputs and internal joint loading, the proposed framework enables the targeted suppression of stress on specific articular structures—most notably the ACL—without compromising the mechanical demands of the exercise. These findings support the development of next-generation electromechanical training devices capable of adapting resistance in real time to optimize joint loading profiles. Such advancements may provide a theoretical basis for future development of technologies aimed at enhancing the safety and efficacy of rehabilitative interventions and athletic conditioning programs, pending experimental validation.

## Data Availability

The raw data supporting the conclusions of this article will be made available by the author on request. The data are not publicly available due to the absence of raw datasets, as the study is purely analytical and all results are derived from the equations explicitly presented in the manuscript.

## Abbreviations

The following abbreviations are used in this manuscript:

ACL	Anterior cruciate ligament
OKC	Open kinetic chain
PCL	Posterior cruciate ligament
ROM	Range of motion

## Appendix A

Equivalence Between Multiple Resistance Forces and a Single Resultant Force

In practical implementations of electromechanical strength machines, the external resistance opposing the movement does not necessarily need to be generated by a single force vector applied at a unique point. More generally, the resisting action can be produced by the combined effect of multiple external forces, each characterized by its own magnitude, direction, and point of application.

Let ${\vec{R}}_{1}$ and ${\vec{R}}_{2}$ denote two resistance forces acting on the moving limb segment, with different orientations and application points. For instance, ${\vec{R}}_{1}$ may be applied perpendicular to the longitudinal axis of the limb segment ($a_{\mathrm{long}}$), while ${\vec{R}}_{2}$ may be applied parallel to $a_{\mathrm{long}}$. Such a configuration may be advantageous from a technological or control perspective, allowing independent modulation of force components with distinct mechanical effects.

According to the principles of rigid-body mechanics, each force can be freely translated along its own line of action without altering its mechanical effect on the system. Consequently, ${\vec{R}}_{1}$ and ${\vec{R}}_{2}$ may be displaced along their respective lines of action until these lines intersect at a common point. At this intersection point, the two forces can be replaced by their vector sum, $\vec{R}={\vec{R}}_{1}+{\vec{R}}_{2}$, without changing the resultant force or moment acting on the limb.

The resulting force vector $\vec{R}$ can, in turn, be translated along its line of action until this line intersects the longitudinal axis of the limb segment, $a_{\mathrm{long}}$. This final step reduces the system to an equivalent single-force representation in which a unique resistance force $\vec{R}$ is applied at a point P located on $a_{\mathrm{long}}$, exactly as assumed in the mechanical model illustrated in Figure 1.

This equivalence demonstrates that the analytical framework developed in the present study is not restricted to resistance generated by a single force element. Instead, it remains valid for more complex resistance-generation strategies involving multiple actuators or force components acting on the limb. This observation justifies the generality of the single-force formulation adopted in the analytical model.

Practical Implementation Considerations

The resistance modulation described above can be achieved using multiple independently controlled linear actuators or cable-driven mechanisms arranged to generate force components with programmable magnitude and orientation. In a leg extension configuration, for example, an angular sensor mounted on the resistance lever can continuously determine the current joint angle, while a linear position sensor allows a dedicated actuator to adjust the point of application of the resistance along the anterior profile of the tibia according to the instantaneous angular position.

In this configuration, one actuator can translate the resistance pad along the tibial surface while simultaneously modulating the magnitude of the applied force, thereby enabling independent control of both the tibiofemoral shear component and the resistance moment. In parallel, an additional force-generating element—such as an instrumented foot platform—may independently regulate the axial traction/compression component acting along the limb without altering the tibiofemoral shear component or the resistance moment.

By continuously regulating actuator outputs through closed-loop control based on real-time kinematic feedback, the system can maintain a prescribed torque profile while simultaneously adjusting the magnitude, direction, and effective application point of the resultant resistance force. Therefore, the adaptive strategy described in this study does not rely on a single actuator capable of arbitrarily changing force direction or application point, but can be realized through coordinated control of multiple force-generating elements whose mechanical equivalence reduces to the single-force formulation adopted in the analytical model (see previous section: Equivalence between multiple resistance forces and a single resultant force).

Actuator and Sensor Specifics

Real-time adaptive electromechanical strength machines capable of modulating resistance parameters during movement have been experimentally investigated in previous work [19]. In such systems, linear and angular positions are measured using high-resolution encoders integrated within the actuation mechanism, typically providing linear resolutions on the order of 10 µm and angular resolutions of approximately 0.01°. These specifications are sufficient to ensure accurate estimation of velocity and acceleration through numerical differentiation while remaining consistent with the bandwidth of voluntary human movement, which is dominated by low-frequency components, with the vast majority of signal power typically contained below approximately 15 Hz, as consistently demonstrated in biomechanical frequency analyses of human movement ([20], pp. 68–73), [41,42]. Sampling frequencies in the range of 100–200 Hz are typically employed to provide adequate oversampling relative to biomechanical signal content and to ensure stable closed-loop control. Higher acquisition rates or sensor resolutions are generally unnecessary, as they do not increase biomechanically meaningful information and may instead amplify measurement noise or mechanical vibration artifacts. In such systems, the total sensing-to-actuation delay typically remains below 25 ms, well below the characteristic time scale of voluntary human movement.

In these systems, resistance actuators commonly operate with available power levels as high as 1–2 kW to guarantee smooth velocity tracking, reliable power computation, limited control delay, and stable real-time modulation of resistance magnitude and application point during dynamic exercise. From a control and actuation standpoint, torque modulation resolutions on the order of 1 Nm are sufficient to ensure smooth and continuous resistance variation under typical operating loads. These characteristics indicate that the adaptive resistance conditions analyzed in the present framework are compatible with the technical capabilities of existing electromechanical strength devices.

## Appendix B

Reference Ranges of ACL Loading and Their Relation to the Present Mechanical Model

To facilitate the clinical interpretation of the proposed analytical framework, it is useful to contextualize the calculated anterior shear joint reaction forces with respect to experimentally reported ACL loading levels. Cadaveric biomechanical studies have shown that the ultimate tensile strength of the intact ACL in young adults is approximately 2160 N [43,44], with a marked age-related reduction to values near 650 N in elderly specimens [44]. These values represent structural failure thresholds rather than physiological operating conditions.

In contrast to structural failure loads, in vivo and model-based measurements demonstrate that ACL forces during physiological activities remain substantially lower. Model-based estimations during walking suggest that ACL forces typically reach peak values within the range of approximately 300 N, occurring in early stance at the moment of contralateral toe-off [45]. During dynamic seated leg extension without external resistance, ACL loading has been estimated to be approximately 140–170 N [46]. More demanding dynamic activities, such as landing and change-of-direction tasks, considerably increase ACL loading; for example, during a running plyometric-type maneuver involving single-leg landing followed by rapid deceleration, ACL tensile forces of approximately 1300 N have been reported [47].

Importantly, the ACL has been shown to provide approximately 86% of the total restraining force against anterior tibial translation [30]. This finding establishes a direct mechanical link between the anterior shear force component calculated in the present model and the load borne by the ACL. Under the assumptions adopted in this study, ACL loading may therefore be estimated as approximately 0.86 times the computed anterior shear component. This conversion enables resistance parameters to be prescribed relative to experimentally reported physiological ACL loading ranges. In practical terms, resistance settings may thus be selected to correspond to a desired fraction of physiologically observed ligament loads (e.g., comparable to walking-level loading in early rehabilitation or progressively increased during advanced strengthening phases).

Therefore, the present model provides a mechanistic framework that may help relate adjustable resistance parameters in electromechanical devices to clinically meaningful ligament loading targets, potentially supporting individualized modulation based on anthropometry, movement kinematics, and stage of recovery. These considerations should be interpreted as analytical projections rather than prescriptive clinical guidelines, and experimental validation will be required to translate the present theoretical framework into practical rehabilitation or training protocols.
